# Fixation of the Membrane during Matrix-Induced Autologous Chondrocyte Implantation in the Knee: A Systematic Review

**DOI:** 10.3390/life12111718

**Published:** 2022-10-27

**Authors:** Filippo Migliorini, Raju Vaishya, Andreas Bell, Christian D. Weber, Christian Götze, Nicola Maffulli

**Affiliations:** 1 Department of Orthopaedic, Trauma, and Reconstructive Surgery, RWTH University Hospital, 52074 Aachen, Germany; 2 Department of Orthopaedic and Trauma Surgery, Eifelklinik St.Brigida, 52152 Simmerath, Germany; 3 Department of Orthopaedics, Indraprastha Apollo Hospitals Institutes of Orthopaedics, New Delhi 110076, India; 4 Department of Orthopaedic, Auguste-Viktoria-Klinik, Universitätsklinikum Ruhr-Universität Bochum, 32545 Bad Oeynhausen, Germany; 5 Department of Medicine, Surgery and Dentistry, University of Salerno, Via S. Allende, 84081 Baronissi, Italy; 6 School of Pharmacy and Bioengineering, Keele University School of Medicine, Thornburrow Drive, Stoke on Trent ST5 5BG, UK; 7 Queen Mary University of London, Barts and the London School of Medicine and Dentistry, Centre for Sports and Exercise Medicine, Mile End Hospital, 275 Bancroft Road, London E1 4DG, UK

**Keywords:** chondral defect, knee, mACI, membrane, scaffolds

## Abstract

Introduction: It is unclear whether the type of membrane used for matrix-assisted autologous chondrocyte implantation (mACI) influences results. A systematic review was conducted to investigate the midterm results of the three most common types of membrane fixation for mACI. Methods: This systematic review was conducted according to the 2020 PRISMA checklist. PubMed, Google Scholar, Embase, and Scopus online databases were accessed in August 2022. All the prospective clinical trials reporting outcomes of mACI in the knee were considered. Studies that describe the modality of membrane fixation (glued, glued, and sutured, no fixation) used for mACI were eligible. Studies that conducted a minimum of 12 months of follow-up were considered. The outcomes of interest were the Tegner Activity Scale and International Knee Documentation Committee (IKDC) score. The rate of failure and revisions were also collected. Results: Data from 26 studies (1539 procedures; 554 of 1539 (36%) were women) were retrieved. The mean follow-up was 42.6 (12 to 84) months. No difference between the groups was found in terms of mean duration of symptoms, age, BMI, gender, and defect size (P > 0.1). No difference was found in terms of the Tegner score (P = 0.3). When no fixation was used, a statistically significant higher IKDC compared to the other groups (P = 0.02) was evidenced. No difference was found in the rate of failure (P = 0.1). The no-fixation group evidenced a statistically significant lower rate of revisions (P = 0.02). Conclusions: No membrane fixation for mACI in the knee scored better than the fastening techniques at the midterm follow-up.

## 1. Introduction

Symptomatic chondral defects of the knee are common in active individuals [1,2]. Chondral defects are debilitating and may lead to retirement from sports [3]. Given its avascular, alymphatic, and hypocellular features, and its low metabolic activity, cartilage exhibits poor healing potential [4,5]. Regardless of the aetiology, the process of healing generally follows three phases: inflammation, proliferation, and remodelling [6,7]. Complex biochemical signalling patterns and interactions between cells, cytokines, and the environment characterise each phase. In hyaline cartilage, this process often is not able to restore healthy tissue, and residual defects are frequent [8,9]. Focal chondral defects up to 1.5 cm^2^ can be treated arthroscopically using the microfractures technique [10,11,12]. For bigger defects, several surgical strategies have been introduced. Autologous chondrocyte implantation (ACI)m a two-step strategy, has been widely performed in patients with focal chondral defects of the knee. During the first procedure, chondrocytes are harvested from a non-weight-bearing zone of the knee. These chondrocytes are then cultured and expanded [13]. In a second surgery, the lesion is debrided and chondrocytes are delivered. The modality of chondrocyte delivery and membrane fixation in ACI changes within three different generations. In the first generation (pACI), the expanded autologous chondrocytes were injected under a periosteal flap sutured over the defect [14]. PACI was burdened by a high rate of hypertrophy, which was attributed to the use of the periosteal flap [15,16,17,18,19,20]. To overcome this complication, collagen-membrane cover ACI (cACI) was introduced, substituting the periosteal flap with a resorbable membrane [21,22]. cACI evolved in matrix-induced autologous chondrocyte implantation (mACI), in which the harvested and cultured autologous chondrocytes are seeded directly on a biodegradable scaffold, either a collagen type I/III matrix or hyaluronic matrix membrane [23]. The loaded matrix allows chondrocytes expansion and is delivered into the chondral defect in the second step of surgery. Compared to the previous generation, mACI allows less invasive approaches (mini-arthrotomy or arthroscopy), avoid graft sutures, and shorter surgical time [24,25]. However, though mACI has been widely performed, there are still controversies. The membrane used in mACI can be fixed in the chondral defect with fibrin glue or can be left without fixation. Some authors, in addition to the fibrin glue, used fine sutures to secure the membrane to the healthy perilesional borders. However, whether to use glue, glue, and suture, or leave the membrane in situ with no fixation is still debated, and no systematic review has compared these methods. The present systematic review compared these procedures of membrane fixation for mACI in the knee. The various membrane delivery systems (glued, glued, and sutured, no fixation) were compared at the midterm follow-up. The outcomes of interest were to compare patient-reported outcome measures (PROMs) and complications between the three membrane delivery strategies. 

## 2. Materials and Methods

### 2.1. Search Strategy

The present systematic review followed the Preferred Reporting Items for Systematic Reviews and Meta-Analyses: the 2020 PRISMA checklist [26]. The PICOT algorithm was conducted as follows:

- P (Problem): focal chondral defect of the knee;
- I (Intervention): mACI;
- C (Comparison): glued, glued & sutured, no fixation;
- O (Outcomes): PROMs and complications.
- T (Timing): ≥ 12 months follow-up.

### 2.2. Data Source and Extraction

The literature search was conducted by two authors (F.M & C.D.W.) independently. PubMed, Google Scholar, Embase, and Scopus online databases were accessed in August 2022. The following keywords were used in combination: *knee AND chondral OR cartilage OR articular OR chondropathy AND damage OR defect OR injury AND matrix-induced autologous chondrocyte implantation OR mACI AND glued OR fibrin OR fixation OR membrane.* The initial screening was conducted by the same authors separately. The full text in PDF form of the selected studies was accessed and downloaded. The bibliographies of the full-text studies were screened by hand by the same investigators. In case of incongruity, all disagreements were debated, and the final decision was taken by a third senior author (N.M.). 

### 2.3. Eligibility Criteria

All the clinical studies which investigated the outcomes of mACI in the knee were accessed. Articles in English, Italian, Spanish, German, and French were included. Prospective studies levels I to II of evidence, according to the Oxford Centre of Evidence-Based Medicine [27], were eligible. Studies reporting less than 5 procedures were not included. Animals or in vitro studies were not considered. Only studies that clearly stated the fashion of the membrane delivery (glued, glued & sutured, no fixation) were included. Only studies that conducted a minimum of 12 months of follow-up were considered. Studies which augmented mACI with other procedures (e.g., mesenchymal stem cells) were not eligible. Missing quantitative data on even one outcome of interest warranted the exclusion from the present investigation. 

### 2.4. Outcomes of Interest

Data extraction was conducted by two authors (F.M & C.D.W.) independently. The generalities of the included studies (author and year, journal of publication, study design) and information on the patient demographic at baseline were collected (sample size, mean BMI, mean age, mean duration of the symptoms prior to surgery, length of the follow-up, percentage of women). Data on the International Knee Documentation Committee (IKDC) [28], the Tegner Activity Scale [29], rate of failures, and revisions at the last follow-up were collected. 

### 2.5. Methodology Quality Assessment

Two authors (F.M & C.D.W.) independently assessed the quality of the included studies using the risk of bias graph tool of the Review Manager Software (The Nordic Cochrane Collaboration, Copenhagen). The risk of selection, detection, attrition, and other bias was quantified.

### 2.6. Statistical Analysis

The statistical analysis was executed by the main author (F.M.) using IBM SPSS Version 25. Continuous data were analysed using the arithmetic mean, standard deviation (SD), and standard error (SE). The odd ratio (OR) effect measure was used for dichotomic data. The confidence interval (CI) was set at 95% in all the comparisons. Analysis of variance (ANOVA) and χ^2^ was performed for continuous and dichotomic data, respectively. Values of P < 0.05 were considered statistically significant. 

## 3. Results

### 3.1. Search Result

**Figure 1:**
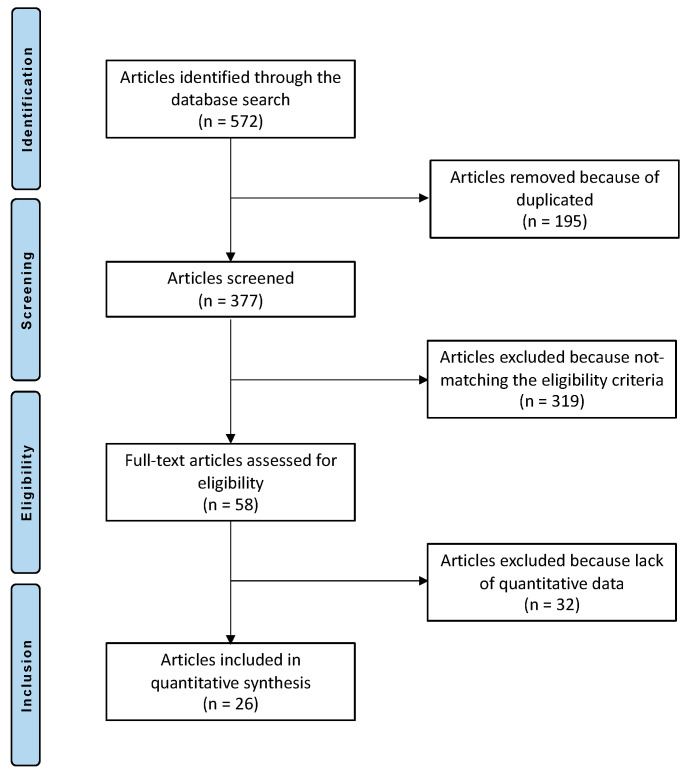
PRISMA Flow chart of the literature search.

572 articles resulted from the initial literature search. Of them, 195 were duplicates. A further 351 articles were excluded as they did not match the eligibility criteria: not focused on mACI (N = 190), not stating the type of fixation (N = 70), low level of evidence (N = 36), missed quantitative data of interest (N = 32), augmented with cell therapies (N = 11), uncertain results or methods (N = 10), language limitations (N = 2). This left 26 studies for the present investigation: 7 RCTs and 19 not randomised prospective studies. The flow chart of the literature search is shown in Figure 1.

### 3.2. Methodological Quality Assessment

**Figure 2:**
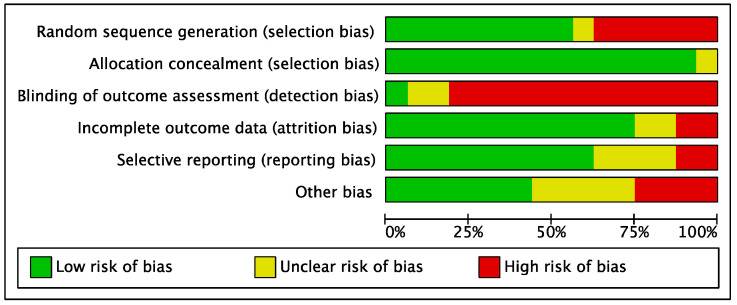
Cochrane risk of bias graph.

For the methodological quality assessment, the Cochrane risk of bias graph was used. The limited number of included RCTs increased the risk of selection bias. The risk of selection bias of allocation concealment was acceptable. The risk of detection bias was moderate. The risk of attrition and reporting bias was low, as was the risk of another type of bias. Overall, the authors of the present review judged as low the risk of bias per each item, attesting to this study’s good methodological assessment. The risk of bias graph is shown in Figure 2. 

### 3.3. Patient Demographics 

1539 mACI procedures were included. 36% (554 of 1539) of mACI procedures were conducted in women The mean length of symptoms prior to surgery was 62.1 (24.2 to 114) months. The mean age of the patients was 32.7 ± 6.6 years. The mean defect size was 3.9 ± 1.3 cm^2^. The mean BMI is 25.1 ± 1.3 kg/m^2^. The mean length of the follow-up was 42.6 (12 to 84) months. There was between-group comparability at baseline in mean age, mean defect size, mean BMI, number of women, and mean length of symptoms (P > 0.1). The generalities and baseline demographic of the studies included in the present systematic review are shown in Table 1. 

### 3.4. Outcomes of Interest

The Tegner score (P = 0.3) resulted similarly at the last follow-up. The no-fixation group evidenced statistically significant lower IKDC compared to the other groups (P = 0.03). The results of PROMs are shown in greater detail in Table 2. 

### 3.5. Complications

The rate of failures (P = 0.1) resulted similarly at the last follow-up. The no-fixation group evidenced statistically significant lower rates of revisions (P = 0.02). The rate of complications is shown in greater detail in Table 3. 

## 4. Discussion

According to the main findings of the present systematic review, no membrane fixation for mACI in the knee scored better than any of the fixation techniques at midterm follow-up. 

mACI is routinely used in the surgical management of focal chondral defects of the knee. Studies must be performed not only to improve and develop the therapeutic principles upon which surgical strategies are based but also to minimize iatrogenic damage, excluding surgical manoeuvres that can compromise the healing process of cartilage. In this context, given the reduced healing capability of the cartilage, suturing of the membrane to the surrounding tissues is questionable. Suturing allows a more stable membrane, but it produces partial-thickness lesions of the articular cartilage. These fissures may not heal and enlarge with time [54,55]. Initially, it was believed that all the membrane procedures were to be fixed using sutures [56]. Hunzinker et al. [57], to establish the potential damage of sutures in cartilage, sutured the surrounding articular cartilage of large, partial-thickness trochlear defects in 18 adult goats. The perisutural area underwent histological, histochemical, and histomorphometrical analysis: suturing induced severe local cartilage impairment which may lead to pain, reduced healing, and premature osteoarthritis [57]. Fibrin glue has been widely employed given its biological sealing, haemostatic and adhesive proprieties [58,59]. Its primary use is as a biological sealant, and it also promotes chondrocytes migration and proliferation [60,61,62]. Mainly through the action of thrombin, fibrin glue promotes a variety of cellular responses, increasing cell migration, proliferation, and survival [63,64]. Fibrin glue promotes osteochondral scaffold fixation and cartilage regeneration [65,66,67], but the results are unpredictable. Indeed, results from the present work clearly demonstrated that no fixation achieves better clinical scores and a lower rate of revision. Even if not statistically significant, the number of failure events was also lower in the no-fixation group. However, the impact of fibrin glue addition on chondrocyte migration and proliferation has not yet been clarified. A recent in vitro study evaluated chondrocyte migration and proliferation with or without fibrin glue application in porcine-derived collagen I/III membrane commonly employed in mACI (Cartmaix, Matricel GmbH, Herzogenrath, Germany) [68]. The no-fibrin group demonstrated greater migration of the cells within the membrane at weeks one, two, and three, and greater proliferation at weeks four, six, and eight [68]. These results should encourage the researcher to conduct additional comparative trials, with MRI and histological evaluation.

The present study has several limitations. We were unable to identify clinical investigations which compared the two fixation modalities. Therefore, a formal meta-analysis was not possible to conduct. None of the included studies aimed to evaluate membrane fixation, introducing biases and impairing the validity of the results of the present study. Only studies in which membrane fixation was performed in the same fashion were included. Given the lack of quantitative data, it was not possible to include the outcome of the isolated membrane suture in the analyses. Authors who perform sutures occasionally were not considered eligible. We must acknowledge that none of the included articles aimed to compare directly the effect of membrane fixation on cartilage healing. This may lead to biased results, and represents the most important limitation of the present study. The limited number of included articles and procedures also represents a limitation. All the analyses were included irrespective of membrane composition (hyaluronic acid or collagen), aetiology (traumatic, osteochondritis dissecans), and surgical exposure (arthroscopy, mini-open, arthrotomy). Studies investigating mACI augmented with cell therapies were not included. Recently, several clinical trials investigated MSCs augmentation for the regeneration of cartilage in patients with symptomatic chondral defects of the knee [30,69,70,71,72]. MSCs are believed to hold great potential in several ailments of the musculoskeletal system [73,74,75]; however, their clinical application is still challenging, and a deeper understanding of their biology is necessary to optimize tissue neogenesis. Between studies differences in surgical procedures were evident. Two studies used membrane-assisted autologous chondrocyte transplantation (mACT) [42,43]. In mACT, chondrocytes are cultivated and expanded into a membrane in the same fashion as mACI and transplanted in the defect with custom-made instruments in a full-arthroscopic fashion [40,41]. Some studies combined results of primary and revision settings. Most studies reported data over multiple locations, often mixing condylar, tibial, trochlear, and patellar defects. Finally, some studies reported data from mACI combined with other surgical interventions, including meniscal procedures, tibial tubercle transfers, and osteotomies. Given these limitations, the results from the present study should be interpreted with caution. The optimal fixation strategy of the membrane during mACI is still unclear, and future methodologically robust investigations are strongly required. Future studies should validate the results of the present study in a clinical setting.

## 5. Conclusions

No membrane fixation for mACI in the knee scored better than the other membrane fixation techniques at the midterm follow-up.

## Figures and Tables

**Table 1:** Generalities and patient demographic of the included studies.

Author, Year	Journal	Study Design	Follow-up (months)	Membrane Fixation	Procedures (*n*)	Female (%)	Mean Age	Mean BMI
Akgun et al. 2015 [30]	*Arch Orthop Trauma Surg*	RCT	24	Control Group	7	0.5714	32.3	24.1
				Both	7	0.5714	32.7	24.3
Basad et al. 2010 [31]	*Knee Surg Sports Traumatol Arthrosc*	RCT	24	Glued	40	0.38	33	25.3
				Control Group	20	0.15	37.5	27.3
Basad et al. 2014 [32]	*Knee Surg Sports Traumatol Arthrosc*	Non-RCT	60	Glued	25	0.37	32	24
Brittberg et al. 2018 [33]	*Am J Sports Med*	RCT	60	Glued	65	0.38	35	
				Control Group	63	0.33	34	
Cvetanovich et al. 2017 [34]	*Am J Sports Med*	Non-RCT	24	Control Group	12	0.22	17	22.8
			24	Both	11	0.22	17	22.8
			24	Both	14	0.22	17	22.8
Ebert et al. 2012 [35]	*Arthroscopy*	Non-RCT	24	Glued	20	0.5	34	26.6
Ebert et al. 2015 [36]	*Am J Sports Med*	Non-RCT	24	Glued	10	0.2	39	25.8
				Glued	13	0.07	36	25.6
				Glued	9	0.66	38	25.1
				Glued	15	0.53	37	25.3
Ebert et al. 2017 [37]	*Am J Sports Med*	Non-RCT	60	Glued	31	0.51	35	26
Efe et al. 2011 [38]	*Am J Sports Med*	Non-RCT	24	None	15	0.6	26	
Ferruzzi et al. 2008 [39]	*J Bone Joint Surg*	Non-RCT	60	Control Group	48	0.38	32	
				None	50	0.28	31	
Filardo et al. 2011 [40]	*Am J Sports Med*	Non-RCT	84	None	62	0.23	28	
Filardo et al. 2014 [41]	*Am J Sports Med*	Non-RCT	84	None	131	0.35	29	24
Kon et al. 2009 [42]	*Am J Sports Med*	Non-RCT	60	None	40	0.17	29	
				Control Group	40	0.32	31	
Kon el al. 2011 [43]	*Am J Sports Med*	Non-RCT	61	None	22	0.32	46	24.7
			58	Glued	39	0.35	45	25.6
Lopez-Alcorocho et al. 2018 [44]	*Cartilage*	Non-RCT	24	Both	50	0.3	35	
Macmull et al. 2011 [45]	*Int Orthop*	Non-RCT	66	Control Group	24	0.29	16	
				Both	7			
Macmull et al. 2012 [21]	*Am J Sports Med*	Non-RCT	45	Control Group	25	0.8	35	
			35.3	Glued	23	0.61	35	
Marlovits et al. 2012 [46]	*Am J Sports Med*	Non-RCT	60	Glued	24	0.12	35	
Meyerkort et al. 2014 [47]	*Knee Surg Sports Traumatol Arthrosc*	Non-RCT	60	Both	23		42	
Niemeyer et al. 2016 [48]	*Am J Sports Med*	RCT	12	None	25	0.33	33	24.9
				None	25	0.16	34	25.6
				None	25	0.4	34	25.1
Niemeyer et al. 2019 [49]	*Orthop J Sports Med*	RCT	24	None	52	0.36	36	25.7
				Control Group	50	0.44	37	25.8
Saris et al. 2014 [50]	*Am J Sports Med*	RCT	24	Glued	72	0.37	35	26.2
				Control Group	72		33	26.4
Schneider et al. 2011 [51]	*Am J Sports Med*	Non-RCT	30.2	Glued	116	0.42	33	24.5
Schüttler et al. 2019 [52]	*Arch Orthop Trauma Surg*	Non-RCT	60	None	23	0.34		27.8
Siebold et al. 2018 [53]	*Knee Surg Sports Traumatol Arthrosc*	Non-RCT	34.8	None	30	0.36	36	23.8
Zeifang et al. 2010 [20]	*Am J Sports Med*	RCT	24	Both	11	0.45	29	
				Control Group	10	0	30	

**Table 2:** Results of Tegner and IKDC.

Endpoint	Sutured & Glued			Glued			No Fixation			P
	Mean	SD	SE	Mean	SD	SE	Mean	SD	SE	
Tegner	5.50	1.12	0.80	4.50	0.87	0.50	5.10	0.14	0.07	0.3
IKDC	66.73	3.65	1.82	69.75	3.62	1.81	76.34	5.94	2.43	0.03

**Table 3:** Results of complications.

Endpoint	Sutured & Glued	Glued	No Fixation	P
Failure	6/34 (18%)	37/330 (11%)	25/318 (8%)	0.1
Revision	13/84 (15%)	16/165 (10%)	5/154 (3%)	0.02

## Data Availability

The datasets generated during and/or analysed during the current study are available throughout the manuscript.
